# The Neurochemical and Microstructural Changes in the Brain of Systemic Lupus Erythematosus Patients: A Multimodal MRI Study

**DOI:** 10.1038/srep19026

**Published:** 2016-01-13

**Authors:** Zhiyan Zhang, Yukai Wang, Zhiwei Shen, Zhongxian Yang, Li Li, Dongxiao Chen, Gen Yan, Xiaofang Cheng, Yuanyu Shen, Xiangyong Tang, Wei Hu, Renhua Wu

**Affiliations:** 1 Department of Medical Imaging, the 2nd Affiliated Hospital, Medical College of Shantou University, Shantou 515041, China; 2 Department of Rheumatology and Immunology, Shantou Central Hospital, Shantou 515041, China; 3 State Key Laboratory of Organ Failure Research, Department of Biostatistics, Guangdong Provincial Key Laboratory of Tropical Disease Research, School of Public Health and Tropical Medicine, Southern Medical University, Guangzhou 510515, China; 4 WHO Collaborating Centre for Infectious Disease Epidemiology and Control, School of Public Health, Li Ka Shing Faculty of Medicine, The University of Hong Kong, Hong Kong SAR, China; 5 Department of Endocrinology, the 2nd Affiliated Hospital, Medical College of Shantou University, Shantou 515041, China; 6 Department of Medical Imaging, Affiliated Hospital, Jiangnan University, Wuxi, Jiangsu 214062, China; 7 Department of Medical Imaging, Guangzhou Huiai Hospital, Guangzhou 510515, China; 8 Provincial Key Laboratory of Medical Molecular Imaging, Guangdong, Shantou 515041, China

## Abstract

The diagnosis and pathology of neuropsychiatric systemic lupus erythematosus (NPSLE) remains challenging. Herein, we used multimodal imaging to assess anatomical and functional changes in brains of SLE patients instead of a single MRI approach generally used in previous studies. Twenty-two NPSLE patients, 21 non-NPSLE patients and 20 healthy controls (HCs) underwent 3.0 T MRI with multivoxel magnetic resonance spectroscopy, T1-weighted volumetric images for voxel based morphometry (VBM) and diffusional kurtosis imaging (DKI) scans. While there were findings in other basal ganglia regions, the most consistent findings were observed in the posterior cingulate gyrus (PCG). The reduction of multiple metabolite concentration was observed in the PCG in the two patient groups, and the NPSLE patients were more prominent. The two patient groups displayed lower diffusional kurtosis (MK) values in the bilateral PCG compared with HCs (*p* < 0.01) as assessed by DKI. Grey matter reduction in the PCG was observed in the NPSLE group using VBM. Positive correlations among cognitive function scores and imaging metrics in bilateral PCG were detected. Multimodal imaging is useful for evaluating SLE subjects and potentially determining disease pathology. Impairments of cognitive function in SLE patients may be interpreted by metabolic and microstructural changes in the PCG.

Systemic lupus erythematosus (SLE) is an autoimmune disorder that affects multiple organ systems, including the central (CNS) and peripheral nervous systems (PNS). The American College of Rheumatology (ACR)^1^ established case definitions and diagnostic criteria for 19 CNS and PNS syndromes observed in SLE patients, which collectively are referred to as neuropsychiatric systemic lupus erythematosus (NPSLE) syndromes. Neuropsychiatric symptoms vary from overt neurological and psychiatric disorders to more subtle signs such as headaches, mood disorders and defects in cognitive function^2^. Multiple pathogenic factors seem to be involved in the aetiology of NPSLE, including autoantibody production, microangiopathy, intrathecal production of proinflammatory cytokines and atherosclerosis^3^. NPSLE is difficult to diagnose due to its multiple clinical presentations and lack of specific biomarkers or typical lesions that can be detected by currently available diagnostic technology.

Magnetic resonance imaging (MRI) is the best imaging technique for the non-invasive diagnosis of NPSLE, even though a wide range of nonspecific abnormalities have been reported^4^,^5^. The MRI findings in both NPSLE and non-NPSLE patients range from nonspecific small punctate focal lesions in white matter (MW) to more severe large-scale lesions such as cortical atrophy, ventricular dilation, cerebral edema, cerebral infarctions, and intracranial haemorrhage^6^. Because brain pathology in SLE patients is multifactorial and complex, its diagnosis remains challenging. Therefore, a multimodal approach, including both morphological and functional imaging, may be more promising.

Magnetic resonance spectroscopy (MRS) of the human brain *in vivo* allows for non-invasive quantification of biological compounds, such as N-acetylaspartate (NAA), myoinositol (MI), choline-containing compounds (Cho), total creatine (tCr), glutamine (Gln) and glutamate (Glu). It has been widely used in SLE studies to gain insight into the pathology of grey matter (GM) and white matter damage^7^,^8^,^9^,^10^. Previous studies using single-voxel proton MRS (SVS) have demonstrated decreased NAA levels both in NPSLE and non-NPSLE patients. NAA changes were more profound in NPSLE patients than in SLE patients without neuropsychiatric manifestations^11^. However, the majority of these studies used the ratio of metabolites rather than absolute quantification, making it difficult to draw firm conclusions on which of the two metabolites in the ratio results in the difference. In addition, SVS is limited to one specific area of the brain and suffers from the partial volume effect. Unlike SVS, multivoxel proton MRS (MVS) allows us to simultaneously assess a great number of brain regions^12^.

Diffusional Kurtosis Imaging (DKI) is a relatively new imaging method based on the diffusion properties of water molecules, which aims to describe the non-Gaussian aspect of water diffusion^13^,^14^,^15^. The DKI technique is an extension of diffusion tensor imaging (DTI) in that it not only maintains the ability to estimate all the standard diffusion tensor metrics—including axial diffusivity, radial diffusivity, and fractional anisotropy (FA), but also provides an additional non-Gaussian diffusion, termed diffusional kurtosis^16^. With improved accuracy and sensitivity, DKI has been used in several studies, including both pathological and normal conditions such as cerebral infarction, ageing, Alzheimer’s disease, etc^15^,^17^,^18^. However, to our knowledge, no assessment has been performed to investigate the microstructural properties of SLE patients using DKI.

Voxel-based morphometry (VBM) is an automated method used for analyzing both regional differences in cerebral volume and tissue volume differences in the brain, using whole-brain MRI data^19^. In the past few years, VBM has been frequently used for investigating GM decline in the context of neurodegenerative pathologies and healthy ageing^20^,^21^. Cortical atrophy detected by MRI was a very common change in the structural brain of NPSLE patients, just after small punctate focal lesions in white matter^22^. However, until now, few studies have used this automated whole-brain analysis method to investigate changes in brain morphology in SLE patients with and without neuropsychiatric symptoms, showing atrophy in specific regions compared with healthy controls^22^,^23^,^24^,^25^. Furthermore, results from these studies have not always been consistent. The reasons for this may include small or unevenly distributed samples, different methods for image processing and different statistical approaches^26^. In the present study, we evaluated MR images of NPSLE and non-NPSLE patients to examine the volume changes in grey matter using the VBM technique with statistical parametric mapping (SPM8) and diffeomorphic anatomical registration through exponentiated Lie algebra (DARTEL), which provides more accurate segmentation and registration compared with conventional VBM^20^.

The present work was designed to overcome the limitations of previous studies using single MRI technology. We combined MRS, DKI and VBM methods in the same groups of subjects. The purpose of this study is to evaluate the metabolites, microstructural changes and abnormality in the volume of grey matter in SLE patients. We also aim to analyse the correlations between different aspects of brain damage and cognition function. Furthermore, we seek the most clinically useful imaging methods that can distinguish SLE patients with CNS lesions from those without CNS lesions.

## Results

### Demographics and clinical characteristics

Twenty-two NPSLE subjects, twenty-one non-NPSLE subjects and twenty healthy controls (HCs) successfully completed the entire protocol and met the pre-established criteria of spectra quality. The following neuropsychiatric manifestations occurred in 22 NPSLE patients: seizure disorder (n = 8), severe headache (n = 5), stroke (n = 3), peripheral polyneuropathy (n = 2), acute confusional state (n = 1), anxiety (n = 2), and myelitis (n = 1).

The three groups did not differ significantly in terms of age (*F* = 0.123, *p* = 0.884), gender (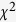 = 0.919, *p* = 0.632) and education (*F* = 1.538, *p* = 0.223). Similarly, differences in Systemic Lupus Erythematosus Disease Activity Index (SLEDAI) scores (*t* = −0.623, *p* = 0.537), disease duration (*t* = −1.683, *p* = 0.101) and cumulative dose of corticosteroids (*t* = 2.419, *p* = 0.128) between NPSLE and non-NPSLE patients were not statistically significant. Mean Mini-mental State Examination (MMSE)^27^ scores (*p* < 0.01) and Montreal Cognitive Assessment (MoCA)^28^ scores (*p* < 0.01) were significantly lower in NPSLE and non-NPSLE subjects compared with HCs, while there were no differences between the two patient groups. The demographic and clinical characteristics of subjects are presented in Table 1.

### MRS

We acquired spectra from the bilateral posterior cingulate gyrus (PCG), dorsal thalamus (DT), lentiform nucleus (LN) and posterior paratrigonal white matter (PWM) in all subjects. Tables 2 and 3 show the differences in all measured metabolite levels from bilateral PCG and DT. The differences in metabolite levels from bilateral LN and PWM can be found in Supplementary Table S1 online. Compared with control subjects, NPSLE patients had significantly lower NAA values in bilateral PCG, DT and left LN (LLN), with mean differences of −1.498 (95% confidence interval (CI): −2.577, −0.419), −1.456 (95% CI: −2.511, −0.401), −1.144 (95% CI: −1.870, −0.418), −0.965 (95% CI: −1.740, −0.191), and −1.421 (95% CI: −2.319, −0.523) for right PCG (RPCG), left PCG (LPCG), right DT (RDT), left DT (LDT) and LLN respectively. The concentrations of tCr were observed to decline in the bilateral PCG and RDT, with mean differences of −1.126 (95% CI: −1.895, −0.356), −1.399 (95% CI: −2.111, −0.686), and −1.109 (95% CI: −1.883, −0.335) for RPCG, LPCG and RDT, respectively. Glx values decreased in RDT and LPCG, with mean differences of −3.407 (95% CI: −5.158, −1.656) and −2.721 (95% CI: −5.031, −0.411), respectively. Cho values decreased significantly in LPCG (mean difference = −0.282, 95% CI: −0.471, −0.093).

Compared with the control subjects, non-NPSLE patients had a lower level of tCr in the bilateral PCG, with mean differences of −1.206 (95% CI: −1.994, −0.418) and −0.775 (95% CI: −1.419, −0.131) for RPCG and LPCG, respectively. NAA values decreased in LLN (mean difference = −0.888, 95% CI: −1.716, −0.060). However, the concentration of Cho was higher in right LN (RLN) (mean difference = 0.295, 95% CI: 0.044, 0.545).

**Figure 1:**
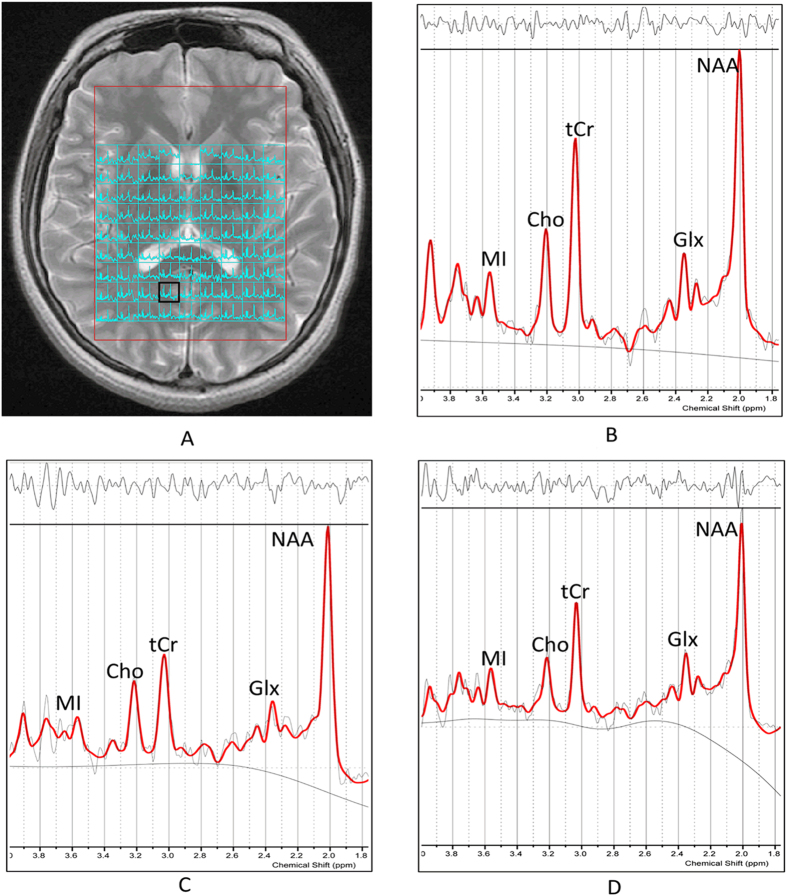
(**A**) Axial view of basal ganglia slice showing the volumes of interest for multivoxel MRS. (**B–D**) Examples of attained spectra of the RPCG by LCModel evaluation of metabolite values: (**B**) HC, (**C**) non-NPSLE patient and (**D**) NPSLE patient. We found NAA decreased significantly in NPSLE patients (*p* < 0.01), followed by non-NPSLE patients (*p* > 0.05), while tCr levels decreased in NPSLE (*p* < 0.01) and non-NPSLE patients (*p* < 0.01) compared with HC. Abbreviations: MRS = magnetic resonance spectroscopy; NPSLE = neuropsychiatric systemic lupus erythematosus; HC = healthy control; RPCG = right posterior cingulate gyrus; NAA = N-acetylaspartate; Cho = choline; tCr = total creatine; MI = myoinositol; Glx = glutamine + glutamate.

Compared with non-NPSLE patients, NPSLE patients had a lower NAA level in LPCG (mean difference = −1.244, 95% CI: −2.196, −0.291) and the Glx level was found to decrease in RDT (mean difference = −1.915, 95% CI: −3.475, −0.355). Figure 1 shows decreased NAA and tCr levels in RPCG in NPSLE and non-NPSLE patients compared with HCs, though there were no statistically significant differences in NAA between non-NPSLE patients and HCs.

### DKI

**Figure 2:**
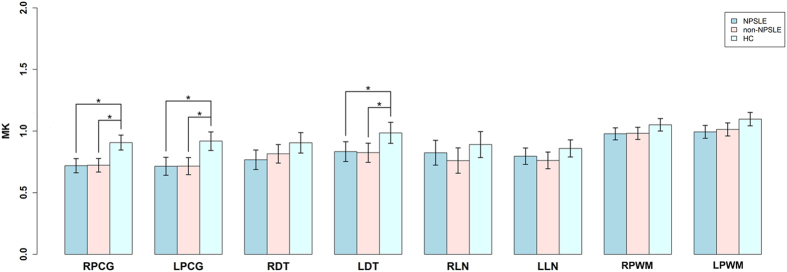
MK values in 8 brain regions in 3 groups. We found that NPSLE and non-NPSLE groups had significantly lower MK values in bilateral PCG (*p* < 0.01) and LDT (*p* < 0.05) compared with the HC group, while the two patient groups did not show differences. ^*^Statistically significant. Abbreviations: MK = diffusional kurtosis; NPSLE = neuropsychiatric systemic lupus erythematosus; HC = healthy control; PCG = posterior cingulate gyrus; DT = dorsal thalamus; LN = lentiform nucleus; PWM = posterior paratrigonal white matter.

The NPSLE and non-NPSLE patients had lower MK values in bilateral PCG and LDT compared with HCs, with mean differences of −0.188 (95% CI: −0.277, −0.099), −0.203 (95% CI: −0.315, −0.092), and −0.151 (95% CI: −0.277, −0.026) for the NPSLE group in RPCG, LPCG and LDT, respectively; and −0.184 (95% CI: −0.267, −0.102), −0.202 (95% CI: −0.305, −0.099), and −0.161 (95% CI: −0.276, −0.045) for the non-NPSLE group in RPCG, LPCG, LDT, respectively. However, we did not observe any difference between NPSLE and non-NPSLE patients (Fig. 2).

### VBM

**Figure 3:**
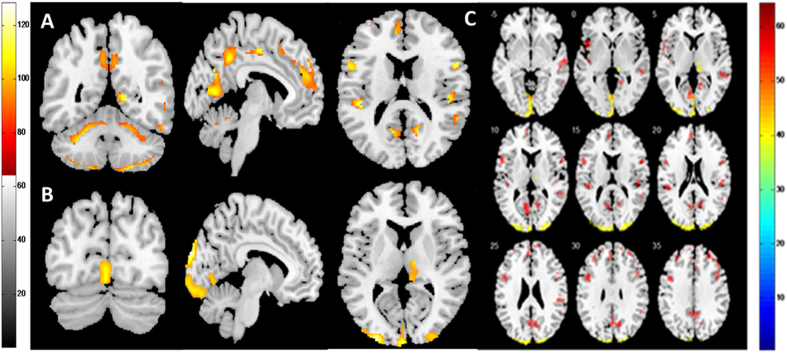
Decreased GM volume in NPSLE patients compared with non-NPSLE patients and HCs. Red and yellow designate differences in GM volume that were statistically significant at *p* < 0.05 after correction for multiple comparisons. (**A**) Coronal, sagittal and axial views of regions with decreased GM in NPSLE patients compared with HCs. (**B**) Coronal, sagittal and axial views of regions with decreased GM in NPSLE patients compared with non-NPSLE patients. (**C**) Axial sections showing details of regions with GM decreases in NPSLE patients. Differences between the HCs and NPSLE patients are shown in red; differences between the non-NPSLE and NPSLE patients are shown in yellow. The figure shows that NPSLE patients had decreased GM volume in the limbic cortex, bilateral frontal lobe, temporal lobe, occipital lobe and right thalamus compared with non-NPSLE patients and HCs. Abbreviations: NPSLE = neuropsychiatric systemic lupus erythematosus; HC = healthy control; PCG = posterior cingulate gyrus; GM = grey matter.

We observed GM reduction in several regions of the brain in NPSLE patients, compared with both non-NPSLE patients and controls (Fig. 3). Compared with controls, NPSLE patients showed GM reduction mainly distributed in the limbic lobe that extended to the posterior cingulate cortex and the bilateral frontal and temporal lobes. Compared with non-NPSLE patients, NPSLE patients showed a GM decline in the bilateral occipital lobe and right thalamus. No significant difference was observed between non-NPSLE patients and controls.

### Correlations between imaging findings and clinical variables

**Figure 4:**
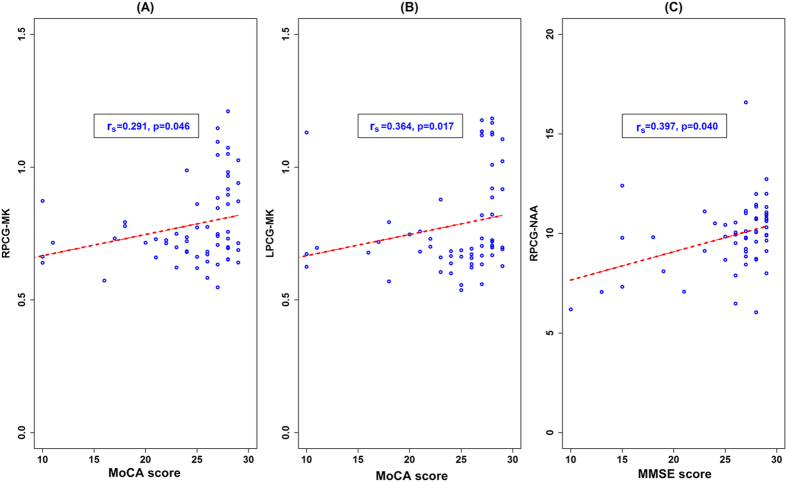
Correlations between imaging metrics and cognitive function scores. The positive correlations between MoCA scores and MK values in RPCG (**A**) and LPCG (**B**). The positive correlations between MMSE scores and MK values in RPCG (**C**). Abbreviations: PCG = posterior cingulate gyrus; MMSE = Mini-mental State examination; MoCA = Montreal Cognitive Assessment; NAA = N-acetylaspartate; MK = diffusional kurtosis.

Among imaging metrics and cognitive scores, positive correlations were observed between MMSE scores and NAA values in RPCG (*r*_*s*_ = 0.397, *p* = 0.040). Positive correlations were also found between MoCA scores and MK values in the bilateral PCG (RPCG: *r*_*s*_ = 0.291, *p* = 0.046; LPCG: *r*_*s*_ = 0.364, *p* = 0.017) (Fig. 4). Between different imaging metrics, consistent declines were observed between tCr and MK in the RPCG in NPSLE patients (*r*_*s*_ = 0.431, *p* = 0.043). No other significant correlations were found between imaging metrics and cognitive scores, nor were they found among the MRS, DKI and VBM measures in the PCG.

## Discussion

MRS was employed to assess changes in the brain metabolites. Compared with HCs, we found that the NAA level decreased in several regions according to multiple volume of interest comparisons, which included the bilateral PCG, DT and LLN in NPSLE patients as well as the LLN in non-NPSLE patients. NAA is found almost entirely in the mitochondria in neural cells, and is also involved in the synthesis of myelin. It has been proposed as a specific marker for viable neurons, axons and dendrites^29^. The present study may reflect a combination of neuronal atrophy, axonal loss, decreased neural metabolism, reduced myelination and loss of dendritic structures in both patients. Apparently, the lesions in the NPSLE patients were more prominent. Our findings were in line with previous reports^7^,^8^,^9^,^30^ and were supported by the results of brain autopsies by Brooks *et al.*^31^. The noteworthy results of our study were the lower tCr in the bilateral PCG in both patient groups, which were different from most of the previous reports, though few of those studies had focused on absolute metabolite concentrations. Cr and PCr exist mainly in glial cells and are part of the high-energy phosphate buffering system^32^. In previous reports, tCr concentrations were assumed to be stable. However, some researchers have realised that concentrations of tCr may vary in different pathological conditions. Brooks *et al.* showed that absolute concentrations of tCr were depressed in NPSLE subjects in either lesional tissue or non-lesional tissue^31^. By matching histopathologic changes via brain-autopsy and metabolite concentrations, he demonstrated that reduced tCr was the marker of acute and chronic brain injury and was related to reduced neuronal-axonal density, the presence of gliosis and inflammation. Gasparovic *et al.* also showed lower tCr in ischaemic tissue^32^. In this study, higher Cho was observed in non-NPSLE patients in RLN compared with HCs. Choline is a marker of myelin turnover that can reflect disease status and cognitive function. To our knowledge, the changes in Cho levels were controversial in previous reports^8^,^31^,^33^. Most studies found that Cho was elevated in NPSLE patients, and its levels correlated with vascular and reactive changes including gliosis, vasculopathy, inflammation and edema^31^. It was surprising that we did not find similar changes in NPSLE patients. On the contrary, decreased concentrations of Cho were found in LPCG in NPSLE patients. The reason for this may be that the majority of NPSLE patients in our group were treated with corticosteroid and immunosuppressive therapy, which suppressed the inflammatory response, and they were in the chronic phase when MRI scans were performed. The loss or damage of neurons may also account for the decreased concentration of Cho. Furthermore, our study showed lower Glx levels in NPSLE patients compared with non-NPSLE patients and HCs in RDT, which was in line with one previous MRS study in which an abnormal insular Gln/Cr level was observed in SLE patients^33^. This change could potentially reflect a loss of glutamatergic neurons or glutamatergic synthesis^34^.

DKI was employed to assess microstructural abnormalities. In previous studies, most researchers evaluated the anisotropic neuroarchitectural orientation of white matter by measuring FA^35^,^36^,^37^,^38^. In contrast to the relatively anisotropic movement of water in WM, water diffusion in GM moves more isotropically^16^. Because DKI is a more comprehensive and sensitive tool to detect microstructural changes in both anisotropic and isotropic tissues^39^, we used DKI to evaluate the changes in GM structures in SLE patients. The principal findings of this study were that MK decreased significantly in the bilateral PCG in both NPSLE and non-NPSLE groups compared with the control group, suggesting a decrease in tissue complexity in the two patient groups. The decrease in MK was associated with degenerative processes leading to neuronal shrinkage and changes in axon and myelin^39^,^40^. As shown by the histological study, the predominant pathologic process in NPSLE patients was small-vessel related^35^. Such inflammation of small vessels in the brain might then lead to vasculopathy and cause focal hypoperfusion or microinfarcts, which subsequently result in neuronal injury and metabolic dysfunction^35^,^37^,^38^. Additionally, therapy-induced CNS changes also played an important role in these changes because most of our patients took corticosteroids or other immunosuppression drugs, which could change the water concentration in the brain^37^. In this study, we found no significant differences between the two patient groups, indicating that non-NPSLE patients, although they were clinically unaffected by neuropsychiatric symptoms, also displayed alteration in microstructural integrity. Because such findings have not previously been reported, further studies are required to corroborate our interpretation.

Our results showed extensive areas of decreased regional GM volume in NPSLE patients compared with non-NPSLE patients and controls, which were consistent with other reports^22^,^24^,^41^. The reduction in GM was mainly distributed in the limbic lobe, occipital lobe, frontal lobe, temporal lobe and right thalamus. Interestingly, we found a decrease in the posterior cingulate of the limbic lobe and right thalamus in NPSLE patients. As we mentioned above, we also found abnormal alterations of brain metabolites and MK values in the PCG and DT in the NPSLE group. The similar discovery in the VBM result provided strong evidence that the damage in these two brain regions may be more prominent than in other regions we evaluated. The underlying neurobiological mechanism may involve the neuronal degeneration process, including neuronal loss, reductions of synaptic spines and reduced numbers of synapses^24^,^42^. However, we observed no differences in GM volume between non-NPSLE patients and HCs, which were not in line with our MRS and DKI results. The reasons for these discrepancies were not clear. One possible explanation could be that cumulative damage of neurons did not induce alteration of brain morphology in non-NPSLE patients.

Interesting, when we explored the relationships between MRS, VBM and DKI data, we found that both metabolism and microstructure in the posterior cingulate region changed noticeably in SLE patients compared with other regions evaluated in this study. Furthermore, we conducted correlation analyses between various measures, e. g., MRS vs DKI, MRS vs VBM, and VBM vs DKI, across subjects in the bilateral PCG. We observed consistently decreased tCr and MK (*r*_*s*_ = 0.431, *p* = 0.043) in RPCG in NPSLE patients. Our findings were in accordance with previous studies in which different methods were used^8^,^43^. Why does PCG change so obviously? One potential explanation is that PCG is adjacent to the circumventricular organs, which have incomplete blood brain barriers, and is therefore vulnerable to inflammatory substances in the bloodstream^44^. Another possible reason relates to the position of the PCG in the border zone between the posterior cerebral artery and anterior cerebral artery^45^. Furthermore, the high rate of metabolism within this region may also account for its vulnerability^45^. In humans, cerebral blood flow and metabolic rate are ~40% greater than average within the PCG^46^. Inflammatory factors and/or immune complexes easily move to the walls of its supplying blood vessels and result in damage to the PCG.

The PCG, a limbic cortical region, plays a key role in cognitive function^45^,^47^. In a wide range of neurological and psychiatric disorders, such as Alzheimer’s disease, autistic spectrum disorder, schizophrenia and depression, the PCG was found to be structurally and functionally abnormal^45^,^48^. Although it was well known that SLE, even without neuropsychiatric features, could display cognitive impairment, PCG dysfunction was notably absent from many reports that studied the relationship between SLE and cognitive function. In the present study, we found lower scores of MMSE and MoCA in both SLE patient groups compared with controls, and the NPSLE group tended to have lower levels than the SLE group, suggesting great impairment in cognitive function within both groups, especially in NPSLE patients. Specifically, we observed significant correlations between cognitive function scores and either NAA or MK values in the PCG. Our study provided evidence of an association between cognitive function and neurometabolic and microstructural abnormalities in this region, which few of studies have specifically addressed. As noted above, it is possible that the dysfunction of PCG may explain, at least in part, the impairments of cognitive function in SLE patients. The thalamus, which has reciprocal connections with the posterior cingulate, is also crucial for memory and directed attention^42^. Abnormal morphology and metabolites in the thalamus observed in our study may also contribute to the cognitive decline in SLE. However, whether its damage is primary or secondary to PCG is still not clear.

The strengths of this study are as follows: (1) We used a multivoxel technique, which yielded data from multiple adjacent voxels covering a large volume of interest in a single measurement and simultaneously generated a wealth of metabolite data. (2) We evaluated the change in metabolites using absolute metabolite concentrations instead of metabolite ratios by LCModel. (3) This study was the first to use the DKI method to show brain microstructural changes caused by SLE. (4) We used a multimodal imaging approach to assess damage to structure and function in SLE patients, which could aid in understanding the pathogenesis of SLE and be potentially useful diagnostically.

Regardless, a number of limitations also exist. A potential limitation of this study was that most patients were treated with corticosteroid and immunosuppressive therapy, which complicated the interpretation of the MRS, DKI and VBM data. Although we have analysed the doses of medication taken by patients and specifically addressed its role as a covariate in the study, we were unable to completely exclude the effects of medication on all three measures. Another limitation was that we did not assess depression in patients. Further studies are ongoing in our institution.

## Conclusion

In conclusion, our study demonstrates that in groups of both NPSLE and non-NPSLE patients, microscopic damage in the brain, including damage to metabolites, microstructure and volume of grey matter, is present and can be detected using a multimodal imaging approach. Of all evaluated brain locations, the area of the posterior cingulate region seems to show the most pronounced alterations and may play an important role in cognitive function. MRS and VBM seem to show the highest usefulness in differentiating the clinical sub-types of CNS involvement from SLE.

## Methods

### Participants

In this study, we evaluated fifty-three consecutive SLE patients and twenty healthy controls (HC) within a 1-year period (from April 2014 to March 2015). Forty patients were recruited from the Department of Rheumatology and Immunology of Shantou Central Hospital; the other 13 patients and all HCs were recruited from the Department of Endocrinology and the Medical Examination Center in our hospital, respectively. All patients were diagnosed with SLE according to the 1997 revised ACR criteria by specialists with experience in this field. The patients fulfilled at least 4 ACR criteria for SLE. The exclusion criteria were as follows: (1) patients unable to undergo MRI, such as subjects with claustrophobia or a pacemaker; (2) patients with past clinical conditions that could cause cerebral atrophy, such as stroke, arterial hypertension, renal insufficiency, diabetes mellitus, alcohol and drug abuse, and cancer; or (3) patients fulfilling the ACR criteria for rheumatoid arthritis, systemic sclerosis, 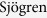 syndrome (primary or secondary), or other connective tissue diseases and drug-induced SLE. Finally, there were forty-three patients included in this study. Among them, 22 patients were diagnosed with NPSLE, defined as at least one or more current or past neuropsychiatric manifestations before inclusion in this study, and 21 patients were diagnosed with non-NPSLE, who had no past or current neuropsychiatric symptoms. HCs did not suffer from any neurologic, psychiatric or systemic illness known to influence brain function, such as serious vascular disease, tumours and epilepsy, and did not have a history of psychoactive medication use. The three groups were matched for age distribution and gender.

The study protocol was designed in accordance with the guidelines outlined in the Declaration of Helsinki and approved by the Research Ethics Committee of the 2nd Affiliated Hospital, Medical College of Shantou University. After a complete description of this study was provided to each subject, informed consent was obtained from the subjects before MR examination.

### Clinical assessments

Data on gender and age, education and disease duration were collected for each patient. Disease duration was defined as the time between the confirmation of SLE and the day of the MRI. The SLEDAI was used to assess current disease status by an experienced rheumatologist and was considered active if scores were greater than 8 points. To evaluate cognitive function, MMSE and MoCA were applied to all participants within 24 hours of MRI scans, whether before or after the scan. Scores ≦24 for MMSE and <26 for MoCA were considered indications of obvious cognitive impairment. Specifically, we analysed the total dose of corticosteroids (COR) and other immunosuppressant medications used since the onset of disease by careful review of medical charts. Doses of oral and intravenous COR were calculated by converting to equivalent doses of prednisone. The cumulative dose of COR used was calculated by summing the daily dosages and multiplying this by the days of treatment.

### Image acquisition and post-processing

All image acquisitions were performed by two experienced neuroradiologists. MRI sequences were performed on a 3.0-T GE (Signa; General Electric Medical Systems) scanner using an eight-channel standard head coil. We used sponge padding and cotton balls to limit head motion and reduce scanner noise. The initial routine MRI and diffusion weighted images, T2-weighted images (T2WI), and the fluid attenuated inversion recovery image (T2FLAIR) were performed to acquire information in the brains of all participants. A T2WI scan (repetition time (TR) = 4,420.0 ms; echo time (TE) = 112.1 ms; 5.0 mm thick; 1.0 mm septation; matrix 512 × 512; field of view (FOV) = 160 × 160 mm) was used for localization of the spectroscopic acquisitions.

MRS was performed with a two-dimensional multivoxel technique using a point-resolved spectroscopy sequence (PRESS) with the following parameters: TE = 35 ms, TR = 1500 ms, number of excitations (NEX) = 1, and phase × frequency = 18 × 18. We chose the basal ganglia level as the volumes of interest (VOIs) for MRS acquisition (see Fig. 1(a)). The total VOIs of the multivoxel section were approximately 160 cm^3^ (10 × 8 × 2 cm: anterior to posterior, left to right, thickness). Water suppression (≥98%) and shimming (linewidth, <15) were automatically performed in all participants using a variable pulse power and optimized relaxation delay scheme. Out-of-field-of-view saturation bands were routinely placed in all MRS examinations. The MRS acquisition scan time was 8 minutes and 12 seconds.

We used SAGE software to correct phase and frequency initially. Metabolite spectra were then analysed using LCModel (LCModel Inc. Canada), a fully automated, commercially available curve-fitting software that uses a least squares analysis method for estimating metabolite concentrations. For each participant, SVS was performed on the position of the posterior cingulate gyrus using the same parameters as MVS, which yielded a reasonable calibration factor. We used these data to calibrate metabolite concentrations. The multivoxel section included the following 8 regions: bilateral PCG, DT, LN and PWM. Metabolite concentrations of NAA, tCr, Cho, MI and Gln + Glu (Glx) were obtained for each voxel. To ensure high-quality data, we excluded absolute metabolite concentrations with standard deviations greater than 20% in the LCModel output.

High-resolution structural MRI scans were taken on each subject using three-dimensional brain volume imaging (3D-BRAVO)^49^ sequences with the following settings: TR = 6 ms, TE = 1.8 ms, FOV = 256 × 256 mm, flip angle = 15 degrees, 1.0 mm thick with no interslice gap, matrix = 256 × 256, NEX = 1, bandwidth 41.67 Hz, and scan time 2 minutes 59 seconds. The whole brain data were acquired in sagittal planes, yielding 168 continuous slices with an acquired voxel size of 1 × 1 × 1 mm.

The SPM8 software package (Wellcome Department of Imaging Neuroscience, London, United Kingdom) based on Matlab 2013a (The MathWorks, Natick, MA, USA) was used to preprocess and analyse MRI data. We segmented the structural images into grey matter, white matter, and cerebrospinal fluid images using a unified segmentation procedure after image-intensity nonuniformity correction. These segmented grey and white matter images were then used to obtain a more accurate inter-subject registration model using DARTEL^50^. This model alternates between computing a group template and warping an individual tissue probability map into alignment with this template, and ultimately creates an individual flow field of each participant. We then spatially normalized each participant’s images to a common template optimized for the population under study, and then smoothed them with an 8-mm full width at half maximum (FWHM) Isotropic Gaussian Kernel. Statistical analysis was performed among three discrete groups, with age, sex, total intracranial volume (TIV) and COR use as covariates, which was a two-sample t-test in SPM8. To correct for multiple comparisons, we used the AlphaSim application (implemented in the REST 1.8, Song *et al.*) with a *p* < 0.05 (corrected, voxel level). Visualization of results was performed using xjview (Functional Imaging Laboratories).

**Figure 5:**
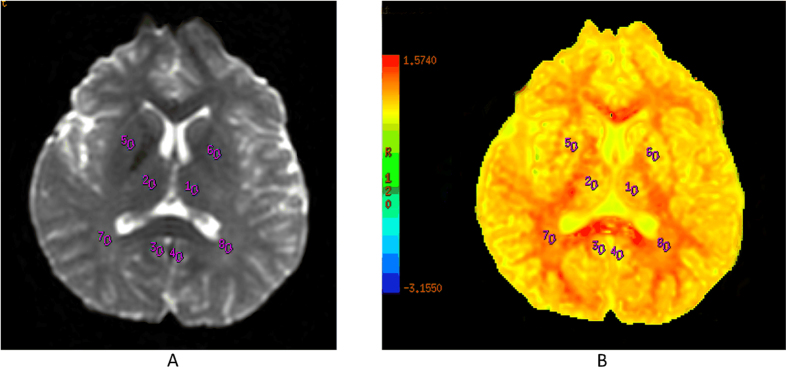
Images of volumes of interest of the bilateral PCG, DT, LN and PWM are depicted in pictures (A,B) for DKI. Abbreviations: DKI = diffusional kurtosis imaging; PCG = posterior cingulate gyrus; DT = dorsal thalamus; LN = lentiform nucleus; PWM = posterior paratrigonal white matter.

DKI image acquisition was performed using an echo planar imaging (EPI) sequence with the following parameters: TR = 4500 ms, TE = 84.1 ms, diffusion gradient pulse duration (δ): 32.2 ms, diffusion gradient separation (Δ): 38.776 ms, FOV = 240 × 240 mm, matrix = 128 × 128, NEX = 1, 5.0 mm thick with no interslice gap, and number of slices = 20. The total scan time was 3 minutes and 5 seconds. Diffusion encoding was applied in 15 directions with three b-values (0, 1000 and 2000 s/mm^2^). DKI data were postprocessed on commercial workstations (GE, ADW 4.6) using Functool software^51^ to generate color-coded and parametric maps of MK. We drew VOIs directly on the MK map for volumetric analysis. VOIs were drawn manually three times with a fixed-in-size (20 mm^2^) ellipse. Corresponding to the MRS, we evaluated 8 different anatomic structures, including the bilateral PCG, DT, LN and PWM (see Fig. 5).

### Statistical analyses

All data were presented as 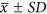. The 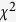 test was used to compare the proportions of males in the three groups. To compare differences in mean values for the SLEDAI, the cumulative dose of corticosteroids, and disease duration between the NPSLE and non-NPSLE groups, we used two independent t tests or Welch’s approach when the assumption that equality of variance for variables in the different groups was rejected using Levene’s test. To compare differences in mean values for age, education, MMSE scores, and MoCA scores among the three groups, we performed analysis of variance (ANOVA) or Welch’s approach. We compared means of absolute metabolite concentrations and MK values after adjusting age and COR dose. If the overall test of the three means for absolute metabolite concentrations and MK values showed statistically significant differences, multiple comparisons of the means of any two groups were made using least significant difference (LSD). The relationships between MRS, DKI, and VBM measures in all evaluated VOIs and cognitive function scores were analysed using Spearman’s rank correlation. We adjusted *p* values using the false discovery rate (FDR) method. The same method was used to examine the correlations among MRS, DKI and VBM measures in PCG. The differences between groups were considered statistically significant at *p* < 0.05 (two-tailed). All analyses were performed with SPSS 21.0 for Windows (SPSS, Chicago, IL) and R 3.2.1 (R Foundation for Statistical Computing, Vienna, Austria).

## Additional Information

**How to cite this article**: Zhang, Z. *et al.* The Neurochemical and Microstructural Changes in the Brain of Systemic Lupus Erythematosus Patients: A Multimodal MRI Study. *Sci. Rep.* **6**, 19026; doi: 10.1038/srep19026 (2016).

## Supplementary Material

Supplementary Information

## Figures and Tables

**Table 1:** Baseline characteristics of subjects.

Characteristic	NPSLE (N = 22)	non-NPSLE (N = 21)	HC (N = 20)	*χ*^*2*^/*F*/*t*	*p*
No. of male (%)	4 (18.2)	2 (9.5)	2 (10.0)	0.919	0.632
Age (yr)^a^	31.270 ± 14.495	29.860 ± 12.273	29.500 ± 7.736	0.123	0.884
Education (yr)	8.500 ± 2.956	9.619 ± 3.201	10.300 ± 3.922	1.538	0.223
MMSE (score)^a^#	24.409 ± 4.777	25.100 ± 5.066	28.450 ± 0.759	11.388	**< 0.001**
MOCA (score)^a^#	21.272 ± 0.504	24.210 ± 4.577	27.600 ± 0.754	14.701	**< 0.001**
SLEDAI (score)	8.381 ± 7.392	7.000 ± 6.532	—	−0.623	0.537
Disease duration (M)^a^	21.857 ± 20.003	12.905 ± 13.935	—	−1.683	0.101
Cumulative dose of COR (gm)	9.356 ± 10.359	5.269 ± 6.284	—	2.419	0.128

^a^Welch’s approach was used for the variable; # Statistically significant for NPSLE vs HC (*p* < 0.01), non-NPSLE vs HC (*p* < 0.05); Abbreviations: NPSLE = neuropsychiatric systemic lupus erythematosus; HC = healthy control; MMSE = Mini-mental State examination; MoCA = Montreal Cognitive Assessment; SLEDAI = Systemic Lupus Erythematosus Disease Activity Index; COR = corticosteroid.

**Table 2:** Absolute concentrations of metabolites (in mmol/L) in bilateral posterior cingulate gyrus and dorsal thalamus.

Metabolites	NPSLE (N = 22)		non-NPSLE (N = 21)		HC (N = 20)		*F*	*p*
	Mean ± SD	Adjusted Mean	Mean ± SD	Adjusted Mean	Mean ± SD	Adjusted Mean		
RPCG								
NAA	9.323 ± 1.174	9.325	9.467 ± 2.512	9.469	10.826 ± 0.980	10.823	4.585	**0.014**
Cho	1.258 ± 0.300	1.257	1.196 ± 0.333	1.237	1.438 ± 0.142	1.425	2.153	0.158
MI	5.445 ± 2.075	5.485	5.147 ± 1.617	5.145	5.660 ± 1.134	5.621	0.360	0.699
Glx	14.940 ± 3.385	14.957	14.267 ± 3.810	14.215	16.417 ± 3.531	16.447	1.654	0.201
tCr	6.203 ± 1.131	6.192	6.121 ± 1.598	6.112	7.297 ± 0.799	7.318	5.947	**0.005**
RDT								
NAA	7.152 ± 1.280	7.206	7.818 ± 0.941	7.823	8.411 ± 0.736	8.350	5.020	**0.010**
Cho	2.053 ± 0.495	2.050	2.107 ± 0.328	2.107	2.145 ± 0.293	2.148	0.260	0.772
MI	5.600 ± 1.418	5.524	5.673 ± 1.279	5.668	5.563 ± 1.467	5.653	0.057	0.944
Glx	12.816 ± 2.752	12.421	14.365 ± 2.217	14.336	15.402 ± 2.375	15.827	7.672	**0.001**
tCr	6.388 ± 1.211	6.316	6.995 ± 1.150	6.989	7.342 ± 0.824	7.425	4.239	**0.019**
LPCG								
NAA	9.757 ± 1.564	9.762	11.013 ± 1.616	11.006	11.219 ± 1.056	11.218	4.668	**0.013**
Cho	1.520 ± 0.291	1.441	1.562 ± 0.317	1.573	1.647 ± 0.221	1.723	4.483	**0.016**
MI	5.924 ± 1.569	5.838	5.510 ± 0.927	5.520	5.795 ± 1.372	5.886	0.438	0.648
Glx	13.870 ± 3.425	13.607	14.176 ± 3.467	14.197	16.047 ± 2.331	16.329	3.154	0.050
tCr	6.563 ± 1.288	6.301	6.901 ± 0.893	6.924	7.435 ± 0.857	7.700	7.769	**0.001**
LDT								
NAA	9.045 ± 0.944	9.071	9.736 ± 1.221	9.737	10.067 ± 1.053	10.036	3.383	0.041
Cho	1.901 ± 0.291	1.877	2.034 ± 0.237	2.034	1.975 ± 0.230	2.000	2.041	0.139
MI	5.290 ± 1.559	5.172	5.494 ± 1.175	5.492	4.466 ± 1.222	4.597	2.087	0.133
Glx	12.725 ± 2.949	12.550	12.438 ± 2.851	12.439	12.242 ± 1.943	12.443	0.010	0.990
tCr	6.488 ± 0.796	6.451	7.001 ± 0.866	7.007	6.922 ± 0.678	6.955	2.959	0.060

Abbreviations: NPSLE = neuropsychiatric systemic lupus erythematosus; HC = healthy control; PCG = posterior cingulate gyrus; DT = dorsal thalamus; NAA = N-acetylaspartate; Cho = choline; tCr = total creatine; MI = myoinositol; Glx = glutamine + glutamate.

**Table 3:** Group differences in metabolite concentration in bilateral PCG and DT.

Metabolites	NPSLE vs non-NPSLE		NPSLE vs HC		Non-NPSLE vs HC	
	Mean difference (95% CI)	*p*	Mean difference (95% CI)	*p*	Mean difference (95% CI)	*p*
RPCG						
NAA	−0.144(−1.221,0.933)	0.970	−1.498(−2.577,−0.419)	**0.007**	−1.354(−2.459, 0.249)	0.057
Cho	—	—	—	—	—	—
MI	—	—	—	—	—	—
Glx	—	—	—	—	—	—
tCr	0.080(−0.688,0.848)	0.835	−1.126(−1.895,−0.356)	**0.005**	−1.206(−1.994,−0.418)	**0.003**
RDT						
NAA	−0.617(−1.265,0.032)	0.062	−1.144(−1.870,−0.418)	**0.003**	−0.527(−1.197,0.142)	0.120
Cho	—	—	—	—	—	—
MI	—	—	—	—	—	—
Glx	−1.915(−3.475,−0.355)	**0.017**	−3.407(−5.158,−1.656)	**<0.001**	−1.492(−3.076,0.093)	0.065
tCr	−0.673(−1.365,0.019)	0.056	−1.109(−1.883,−0.335)	**0.006**	−0.436(−1.150,0.278)	0.226
LPCG						
NAA	−1.244(−2.196,−0.291)	**0.011**	−1.456(−2.511,−0.401)	**0.008**	−0.212(−1.166,0.742)	0.658
Cho	−0.133(−0.306,0.041)	0.131	−0.282(−0.471,−0.093)	**0.004**	−0.149(−0.321,0.022)	0.087
MI	—	—	—	—	—	—
Glx	−0.590(−2.655,1.476)	0.570	−2.721(−5.031,−0.411)	**0.022**	−2.132(−4.225,−0.039)	**0.046**
tCr	−0.623(−1.266,0.020)	0.057	−1.399(−2.111,−0.686)	**<0.001**	−0.775(−1.419,−0.131)	**0.019**
LDT						
NAA	−0.666(−1.355,0.022)	0.058	−0.965(−1.740,−0.191)	**0.015**	−0.299(−1.015,0.417)	0.407
Cho	—	—	—	—	—	—
MI^a^	—	—	—	—	—	—
Glx	—	—	—	—	—	—
tCr	—	—	—	—	—	—

Abbreviations: NPSLE = neuropsychiatric systemic lupus erythematosus; HC = healthy control; PCG = posterior cingulate gyrus; DT = dorsal thalamus; NAA = N-acetylaspartate; Cho = choline; tCr = total creatine; MI = myoinositol; Glx = glutamine + glutamate.
