# Supplementary material for: The Neurochemical and Microstructural Changes in the Brain of Systemic Lupus Erythematosus Patients: A Multimodal MRI Study
Source: Sci Rep. 2016 Jan 13;6:19026. doi: 10.1038/srep19026 (PMC4725825; doi:10.1038/srep19026)
Supplement: Supplementary Information [file srep19026-s1.pdf]

Supplementary Information

The Neurochemical and Microstructural Changes in the Brain of Systemic Lupus Erythematosus Patients: A Multimodal MRI Study

Zhiyan Zhang, Yukai Wang , Zhiwei Shen, Zhongxian Yang, Li Li, Dongxiao Cheng, Geng Yan, Xiaofang Cheng, Yuanyu Shen, Xiangyong Tang, Wei Hu, Renhua Wu

Supplementary Table S1. **Absolute concentrations of metabolites (in mmol/L) in bilateral LN and PWM**

Metabolites	NPSLE (N=22)		non-NPSLE (N=21)		HC (N = 20)		*F*	*p*
	Mean ±SD	Adjusted Mean	Mean ±SD	Adjusted Mean	Mean ±SD	Adjusted Mean		
**RLN**								
NAA	8.043 ±1.372	8.012	7.975 ±1.373	7.971	7.976 ±1.336	8.013	0.006	0.994
Cho**&**	1.751 ±0.292	1.747	1.930 ±0.400	1.930	1.630 ±0.425	1.635	2.983	0.059
MI	5.628 ±2.333	5.778	5.195 ±1.254	5.202	5.026 ±2.297	4.823	0.763	0.472
Glx	13.562 ±3.170	13.575	14.023 ±2.404	14.051	13.705 ±3.725	13.647	0.139	0.870
tCr	6.764 ±1.152	6.771	6.994 ±1.349	6.994	6.570 ±1.416	6.563	0.503	0.607
**LLN**								
NAA#	8.984 ±1.462	8.826	9.373 ±1.266	9.359	10.067 ±1.070	10.247	5.108	**0.009**
Cho	2.251 ±0.365	2.225	2.176 ±0.437	2.174	2.255 ±0.422	2.284	0.348	0.708
MI	5.671 ±1.670	5.808	5.096 ±1.090	5.106	5.442 ±1.754	5.278	1.098	0.341
Glx	15.824 ±7.214	16.325	15.120 ±2.923	15.242	17.295 ±2.777	16.749	0.536	0.588
tCr	8.240 ±1.555	8.206	8.259 ±0.960	8.256	8.599 ±1.398	8.638	0.505	0.606
**RPWM**								
NAA	8.684 ±1.539	8.644	8.925 ±1.755	8.918	9.713 ±0.947	9.764	2.496	0.091
Cho	1.930 ±0.430	1.951	1.861 ±0.247	1.863	2.067 ±0.328	2.041	1.252	0.294
MI	6.008 ±2.187	6.153	5.312 ±1.337	5.323	5.584 ±1.041	5.412	1.456	0.242
Glx	11.107 ±3.332	11.143	11.716 ±2.397	11.719	11.696 ±2.704	11.657	0.220	0.803
tCr	5.532 ±1.105	5.527	5.289 ±1.200	5.289	5.570 ±0.639	5.575	0.450	0.640
**LPWM**								
NAA	8.847 ±1.867	9.027	9.906 ±1.624	9.895	10.320 ±1.257	10.133	2.109	0.131
Cho	2.031 ±0.353	2.023	1.982 ±0.409	1.983	2.164 ±0.263	2.172	1.388	0.258
MI	5.810 ±1.689	6.065	6.132 ±1.301	6.120	5.787 ±1.160	5.517	0.942	0.396
Glx	12.200 ±2.611	12.300	11.031 ±2.354	11.032	11.858 ±2.550	11.748	1.317	0.276
tCr	5.973 ±1.076	6.082	6.268 ±1.049	6.260	5.963 ±0.671	5.850	0.831	0.441

& Statistically significant for non-NPSLE vs HC ($p < 0.05$); **#** Statistically significant for NPSLE vs HC ($p < 0.01$) and non-NPSLE vs HC ($p < 0.05$); Abbreviations: NPSLE = neuropsychiatric systemic lupus erythematosus; HC = healthy control; LN = lentiform nucleus; PWM = posterior paratrigonal white matter; NAA = N-acetylaspartate; tCr = total creatine; Cho = choline; MI = myoinositol; Glx = glutamine + glutamate
